# Colorful Hydrophobic Poly(Vinyl Butyral)/Cationic Dye Fibrous Membranes via a Colored Solution Electrospinning Process

**DOI:** 10.1186/s11671-016-1763-4

**Published:** 2016-12-05

**Authors:** Xu Yan, Ming-Hao You, Tao Lou, Miao Yu, Jun-Cheng Zhang, Mao-Gang Gong, Fu-Yan Lv, Yuan-Yuan Huang, Yun-Ze Long

**Affiliations:** 1 Collaborative Innovation Center for Nanomaterials & Optoelectronic Devices, College of Physics, Qingdao University, Qingdao, 266071 China; 2 Industrial Research Institute of Nonwovens & Technical Textiles, College of Textiles & Clothing, Qingdao University, Qingdao, 266071 China; 3 College of Chemistry and Chemical Engineering, Qingdao University, Qingdao, 266071 China; 4 Department of Mechanical Engineering, Columbia University, New York, NY 10027 USA; 5 Collaborative Innovation Center for Marine Biomass Fibers, Materials & Textiles of Shandong Province, Qingdao University, Qingdao, 266071 China

**Keywords:** Poly(vinyl butyral) (PVB)/cationic dye, Colorful nanofibrous membranes, Hydrophobicity

## Abstract

Colorful nanofibrous membranes have attracted much attention for their visual varieties and various functionalities. In this article, a colored solution electrospinning process was used to fabricate colorful hydrophobic poly(vinyl butyral) (PVB)/cationic dye nanofibrous membranes (NFMs) successfully. The color and morphology of these as-spun nanofibrous membranes have been analyzed by colorimetry, spectroscopy, and scanning electron microscopy (SEM). It is shown that the as-spun colorful PVB-based membranes exhibit excellent level-dyeing property and color stability. Furthermore, the doping of cationic dye and the increase of dye concentration can decrease the diameter of the as-spun colored fibers, which results in better level-dyeing property and higher water contact angle more than 140°. The stained PVB fibrous membranes with excellent level-dyeing property and hydrophobicity are promising in some applications such as textiles, wallpapers, and anticorrosive coating/painting.

**Electronic supplementary material:**

The online version of this article (doi:10.1186/s11671-016-1763-4) contains supplementary material, which is available to authorized users.

## Background

Nowadays, electrospinning (e-spinning) has developed and entered in many fields as a versatile, effective, economical method to fabricate nanofibers [1–4]. The electrospun (e-spun) nanofibers can be collected as nanofiber membrane (NFM) and have been found in various applications such as tissue engineering scaffolds, [5, 6] drug delivery, [6, 7] wound dressing, [8, 9] high efficiency particulate air filter, [10] flexible nano-optoelectronic devices, [11, 12] nanosensors, [12, 13] protective clothing, [14, 15], and so on. However, the typical e-spun NFM usually appears white, which is the common color of polymers and mainly due to the physical phenomenon of light scattering [16, 17].

Recently, efforts have been made to develop colored nanofibrous membranes [17–22]. For coloring e-spun nanofibers, electrospinning-dyeing method is a useful and common process that electrospinning is firstly used to fabricate NFMs and then dyeing the e-spun NFMs by fixing the natural or synthetic dyes onto the NFMs, which can absorb and reflect light at specific wavelengths to give human eyes the sense of color [18–21, 23, 24]. However, the electrospinning-dyeing technique is complex and inefficient, especially for the NFMs with higher surface area and higher ability to scatter more light [19–21].

In addition to the electrospinning-dyeing process, colored solution electrospinning (CSE) which can make the coloration during the formation of nanofibers is also employed [17, 22, 25–27]. In the CSE process, dyes in powder were added to pre-formed polymer solutions and dissolved or dispersed in the solutions by vigorous stirring and then stained nanofibers can be produced by electrospinning process directly [17]. By using this approach, colored PA6 NFMs were prepared by dissolving dyes or by dispersing pigments in the electrospinning solutions [17]. It was mentioned that the coloration approach adopted with organic dyes did not influence the NFM morphology significantly, while the case was contrast for pigment [17]. Moreover, Daneshvar et al. colored e-spun nylon66 nanofiber yarns using the two dyeing method mentioned above, and it was found that the color strength of CSE samples was lower than the electrospinning-dyeing process samples, but their dye levelness was better than the samples of dyeing process [22]. Furthermore, the functional colorful NFMs could be used as PH-sensor and volatile organic compound (VOC)-sensor [25–27]. However, hydrophobicity of colorful nanofibrous membranes via colored solution electrospinning are rarely studied, which are important for practical application.

Poly(vinyl butyral) (PVB) is characterized by high adhesion to glass, excellent mechanical strength, good dispersion for dyes, and light stability, and consequently, it has been extensively used for many applications such as an adhesive interlayer in safety glass, [28] UV indicator, [29, 30], and electron beam irradiation dosimeter [31, 32]. In addition, PVB has both hydrophobic and hydrophilic properties due to its chemical structure with both vinyl butyral group and vinyl hydroxyl group [33, 34]. However, former interests are mostly focused on the hydrophilicity of PVB and its nanocomposite materials [35, 36]. Recently, hydrophobicity of electrospun PVB nanofibrous structures and patterns were investigated and the reported water contact angle (WCA) of electrospun PVB fabrous mat could reach 130°, which was much higher than WCA of the PVB film prepared by solution casting [37, 38]. The high hydrophobicity of e-spun PVB film can be attributed to the nanoscaled rough surface structure of the e-spun PVB nanofabrous mats and demonstrate a possible way to control the drug release from a polymer matrix by modifying the surface with different hydrophobic micro-/nanostructures [38]. However, colorful electrospun PVB NFMs doped with different cationic dyes by the CSE process and their level-dyeing property and hydrophobicity have not been reported yet.

In this study, hydrophobic colorful PVB NFMs were successfully fabricated by the CSE process with PVB/ethanol solutions doping with different cationic dyes. The coloration of the as-spun colorful PVB NFMs was investigated by doping with different dyes and increased dye concentrations. The influences of the coloration upon the morphology of PVB NFMs were also studied. Moreover, the level-dyeing property and hydrophobicity of the colored PVB NFMs were investigated through color and WCA measurements. Furthermore, potential applications of CSE process and as-spun hydrophobic colorful PVB NFMs for color printing were also mentioned.

## Methods

### Preparation of Colorful PVB/Dye NFMs

**Fig. 1:**
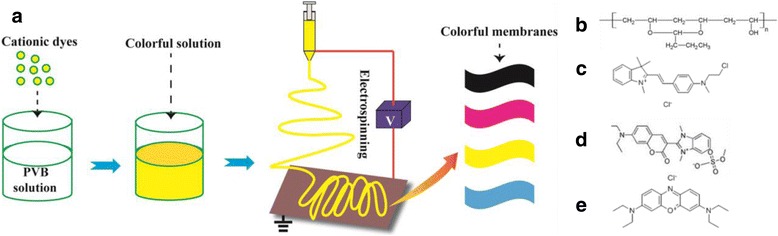
Schematic illustrations of the colored solution electrospinning (CSE) process to prepare colorful nanofiber membranes (**a**). In this process, cationic dyes were added into pre-formed PVB solutions and dissolved in the solutions by vigorous stirring and then cationic dye colored PVB solutions can be e-spun into colorful nanofibers and collected as colorful nanofibrous membranes (NFMs). Molecular structure of PVB (**b**), cationic red dye (X-GRL) (**c**), cationic yellow dye (X-10GFF) (**d**), and cationic blue dye (X-GB) (**e**)

As displayed in Fig. 1a, the polymer solution was prepared by dissolving poly(vinyl butyral) (PVB) (MW ~100,000, Sinopharm Chemical Reagent Co., Ltd., China) in ethyl alcohol at 10 wt% firstly and then stirred thoroughly for 2 h at room temperature. Subsequently, commercial cationic dyes (cationic Red/X-GRL, cationic Yellow/X-10GFF, cationic Blue/X-GB, and cationic black/X-2RL, Winchem Industrial Co. Ltd., China) were dissolved in the prepared PVB solution respectively at 2.5 wt% for 2 h, then the colored PVB precursor solution was obtained. The molecular structures of PVB and cationic dyes X-GRL, X-10GFF, and X-GB were shown in Fig. 1b–e, respectively. However, the molecular structure of cationic dye X-2RL is absent since it is a mixed dye. Similarly, PVB/dye solutions with different dye concentrations were prepared. All the solutions were agitated at room temperature under constant stirring for at least 24 h prior electrospinning. The CSE process was carried out under a high voltage about 15 kV and the distance between the needle and collector about 12 cm at room temperature with collecting time about 30 min. The feeding rate of the solution in the syringe (5 ml) was maintained at 80 μl min^−1^ by using a syringe pump (LSP01-1A, Baoding Longer Precision Pump Co., China).

### Characterization

Photographs of prepared stained NFMs were recorded by Nikon D3 camera with a Nikkor 50-mm f1.2 lense. The absorption spectra of the colorful NFMs were measured using a UV/vis/NIR spectrophotometer (U-4100, Hitachi) with a scan speed of 120 nm min^−1^. The morphologies of the electrospun fibers were characterized by a scanning electron microscope (SEM, TM-1000, Hitachi) and an optical microscope (BX-51, Olympus). Conductivity of the colored solutions was measured by electric conductivity meter (DDS-307, Shanghai Optical Instruments Factory) with cell constant of 0.973. The colorful NFMs were also characterized by Fourier transform infrared spectroscopy (FTIR) using a Thermo Scientific Nicolet iN10 spectrometer, and transmittance data were processed for the wave number range 700–4000 cm^−1^. The static WCAs of all samples prepared were measured on a DataPhysics OCA20 CA system at ambient temperature. Water droplets (2.0 μl) were dropped carefully onto the surface of the PVB/dye composite NFMs. The average WCA value was obtained by measuring the water droplets set at five randomly distributed positions.

### Color Testing

The relative color strength (*K*/*S* value) of colored NFMs at a maximum absorption wavelength λ_max_ were measured by X-Rite Premier 8400 color measurement system (X-Rite, Grand Rapids, MI, USA). The *K*/*S* value can be determined by the Kubelka-Munk equation,

1 $$ K/S={\left(1-R\right)}^2/2R $$

where *K* and *S* are spectral absorption and scattering coefficients, respectively; *R* is the spectral reflectance ratio [39]. And the level dyeing could be evaluated according to the standard deviation σ_*K*/*S*_ of the *K*/*S* values in Eq. 2 [39].

2 $$ {\sigma}_{K/S}={\sqrt{{\displaystyle {\sum}_{i=1}^n}\left[{\left(K/S\right)}_{i,\ {\lambda}_{\max }}-\overline{{\left(K/S\right)}_{i,\kern0.5em {\lambda}_{\max }}}\right]}}^2 $$

3 $$ \overline{K/S}=1/n{{\displaystyle {\sum}_{i=1}^n\left(K/S\right)}}_i $$

where σ_*K*/*S*_ is the standard deviation of the *K*/*S* values of an individual colorful NFM sample under *λ*_max_; *i* = 1, 2, 3,…,n (*n* = 5 was used in this work), refers to the different sites measured on an individual colorful NFM; $\overline{K/S}$ is the arithmetic mean of *K*/*S* values of an individual sample as defined in Eq. 3, also used to assess the color strength of samples [39].

## Results and Discussion

### Color of Prepared NFMs

**Fig. 2:**
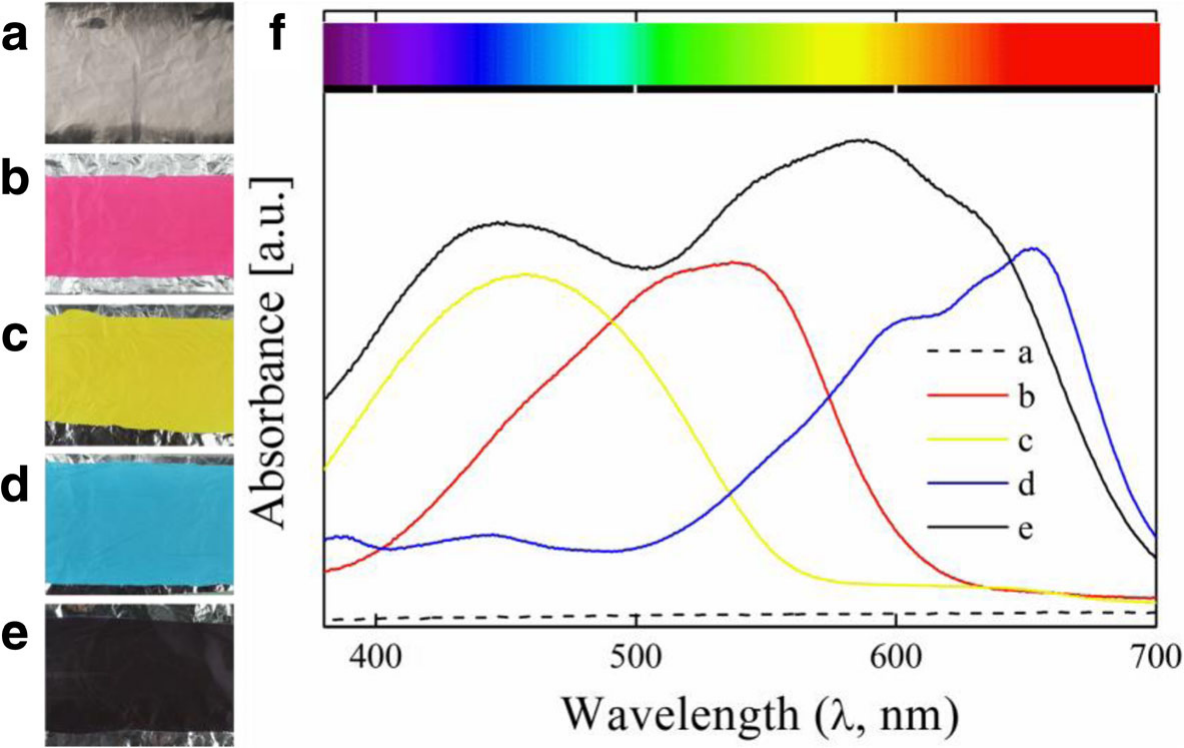
Images (**a**–**e**) are the prepared PVB/dyes colorful NFMs on aluminum foil substrates, where **a** is the pure PVB NFM, **b** PVB/X-GRL NFM, **c** PVB/X-10GFF NFM, **d** PVB/X-GB NFM, and **e** PVB/X-2RL NFM. The absorption spectra of these electrospun stained NFM (**f**) is also measured, where the *inset* color bar shows the color of visible light wavelengths within 380–700 nm

Figure 2a–e shows the images of the obtained pure PVB (Fig. 2a), PVB/X-GRL (Fig. 2b), PVB/X-10GFF (Fig. 2c), PVB/X-GB (Fig. 2d), and PVB/X-2RL (Fig. 2e) NFMs with white, red, yellow, blue, and black colors, respectively. To ensure the color of prepared NFMs, the absorption spectra of different colored NFMs were examined under visible light wavelengths within 380–700 nm. As shown in Fig. 2f, the white pure PVB NFM (Fig. 2a) absorbed few visible light, while the black PVB/X-2RL NFM (Fig. 2e) absorbed almost all the visible lights from 380–700 nm. As for the PVB/X-GRL NFM (Fig. 2b), their exhibits a wide absorption peak from 400–600 nm and the maximum absorbed visible light is under λ_max_ = 573 nm. According to the inset color bar in Fig. 2f, the residual visible lights including the whole red light region and a little purple light region ensure the NFM showing a dark red color. Similarly, the yellow PVB/X-GRL (Fig. 2c) and blue PVB/X-GB (Fig. 2d) NFMs show absorption peaks at 380–550 and 550–700 nm with the maximum absorbed light of λ_max_ = 459 and λ_max_ = 652 nm, respectively. And compared to the color bar, the absorbed wavelengths of PVB/X-GRL and PVB/X-GB rightly ensure the color exhibit as shown in Fig. 2c, d.

### Level-Dyeing Property and Color Stability

To test the level-dyeing property, *K*/*S* values of different sites on an individual colorful NFM sample were measured. It has been suggested that the lower the value of a standard deviation (σ_*K*/*S*_), the better leveling property will be observed on a colorful NFM sample [39]. The parameters of each colorful NFM were concluded in Table. 1. As can be found in Table 1, the standard deviation of the *K*/*S* values of red, yellow, blue, and black NFMs all suggested *σ*_*K*/*S*_ < 0.5, which indicated the prepared PVB colorful NFMs exhibit good level-dyeing property, especially for the PVB/X-GRL NFM. The level-dyeing properties of these PVB/dye composite colored NFMs indicated that CSE process is a useful and efficient way to prepare colorful NFMs, and consistent with former study [22].

**Table 1:** Color testing parameters of different colorful NFMs

Materials	*λ*_max_ (nm)	$\overline{K/S}$	σ_*K*/*S*_
PVB	–	0.0022	3.04 E^−4^
PVB/X-GRL	573	4.74	0.054
PVB/X-10GFF	459	18.78	0.320
PVB/X-GB	652	11.22	0.424
PVB/X-2RL	588	15.28	0.353

**Fig. 3:**
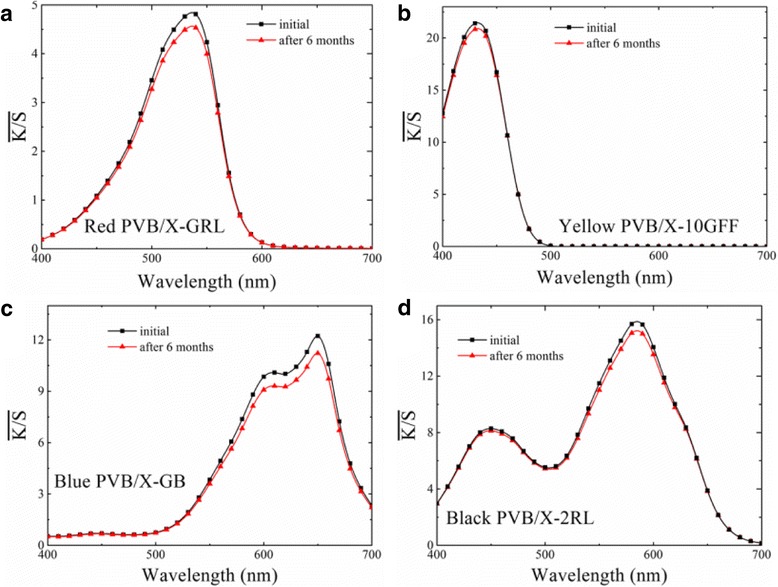
$\overline{K/S}$ curves of prepared colorful NFMs doping with different cationic dyes before and after placement for 6 months corresponding to different colors: **a** red, **b** yellow, **c** blue, and **d** black

Furthermore, to evaluate the stability of the color on PVB nanofiber mats, the $\overline{K/S}$ curves of the prepared composite cationic dye-colored PVB NFMs in red, yellow, blue, and black colors before and after placement for about 6 months under ambient conditions in air were measured and compared, as shown in Fig. 3a–d. It can be found that the $\overline{K/S}$ curves corresponding to the colorful NFMs with different colors changed slightly, indicating that the colors of the PVB/cationic dyes did not vary obviously even after a longtime of 6 months. The sligtly changing of $\overline{K/S}$ may result from the dyes wrapped into the PVB fibers during the CSE process. These results suggest that the as-spun colorful NFMs exhibit good color stability [40], which is significant for the industrial application of colored membranes production by CSE process.

### FTIR Spectra

**Fig. 4:**
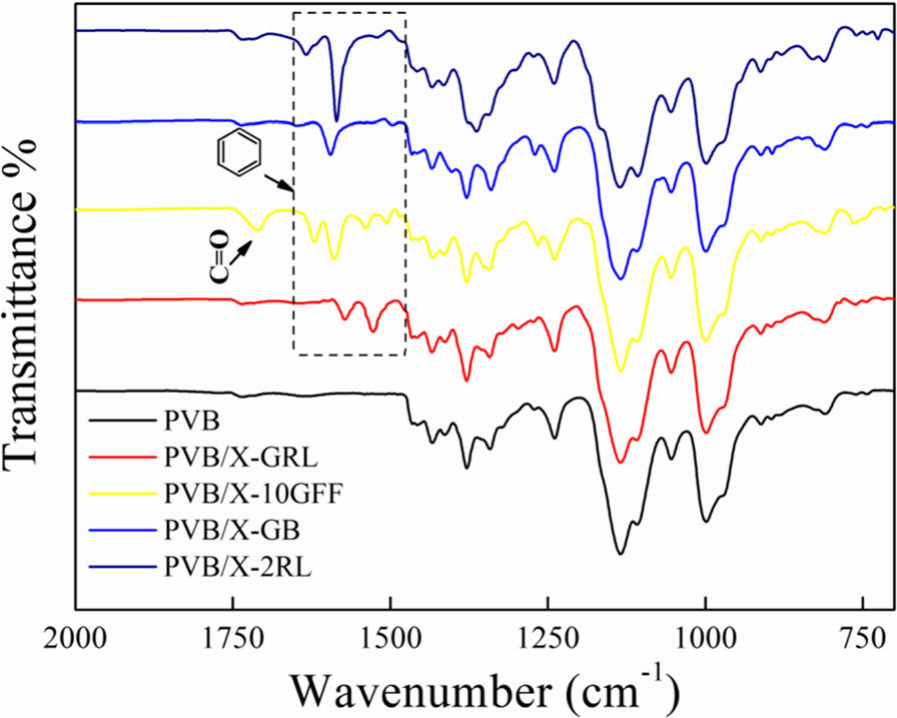
The FTIR spectra of colorful NFMs with different dyes

Figure 4 shows the FTIR spectra of the obtained colorful NFMs. As shown in Fig. 1b–e, all the cationic dyes have phenyl ring groups in their molecular structures. Correspondingly, the FTIR spectra of the colorful NFMs suggest a benzene skeleton vibration in the wavenumber region of 1470–1632 cm^−1^ as displayed in the dashed frame of Fig. 4. Moreover, the PVB/X-10GFF NFM shows an absorption peak at 1709 cm^−1^, which corresponds to the carbonyl group C = O in X-10GFF dye. While for the cationic black (X-2RL), one can only find some possible group from the FTIR spectra such as phenyl ring at wavenumbers 1584 and 1632 cm^−1^. The broad peak observed at 3334 cm^−1^ is due to the –OH stretching of PVB [41], corresponding to the hydrophilic hydroxyl group in PVB. The peaks at 2954 and 2868 cm^−1^ are due to the asymmetric and symmetric –CH2 stretching respectively, and the peak observed around 1433 cm^−1^ is due to –CH2 bending [41], which can be attributed to the hydrophobic group in PVB. Moreover, it is suggested that dye doping shows no significant change in the functional groups among the PVB nanofibers.

### Morphology of Colorful NFMs

**Fig. 5:**
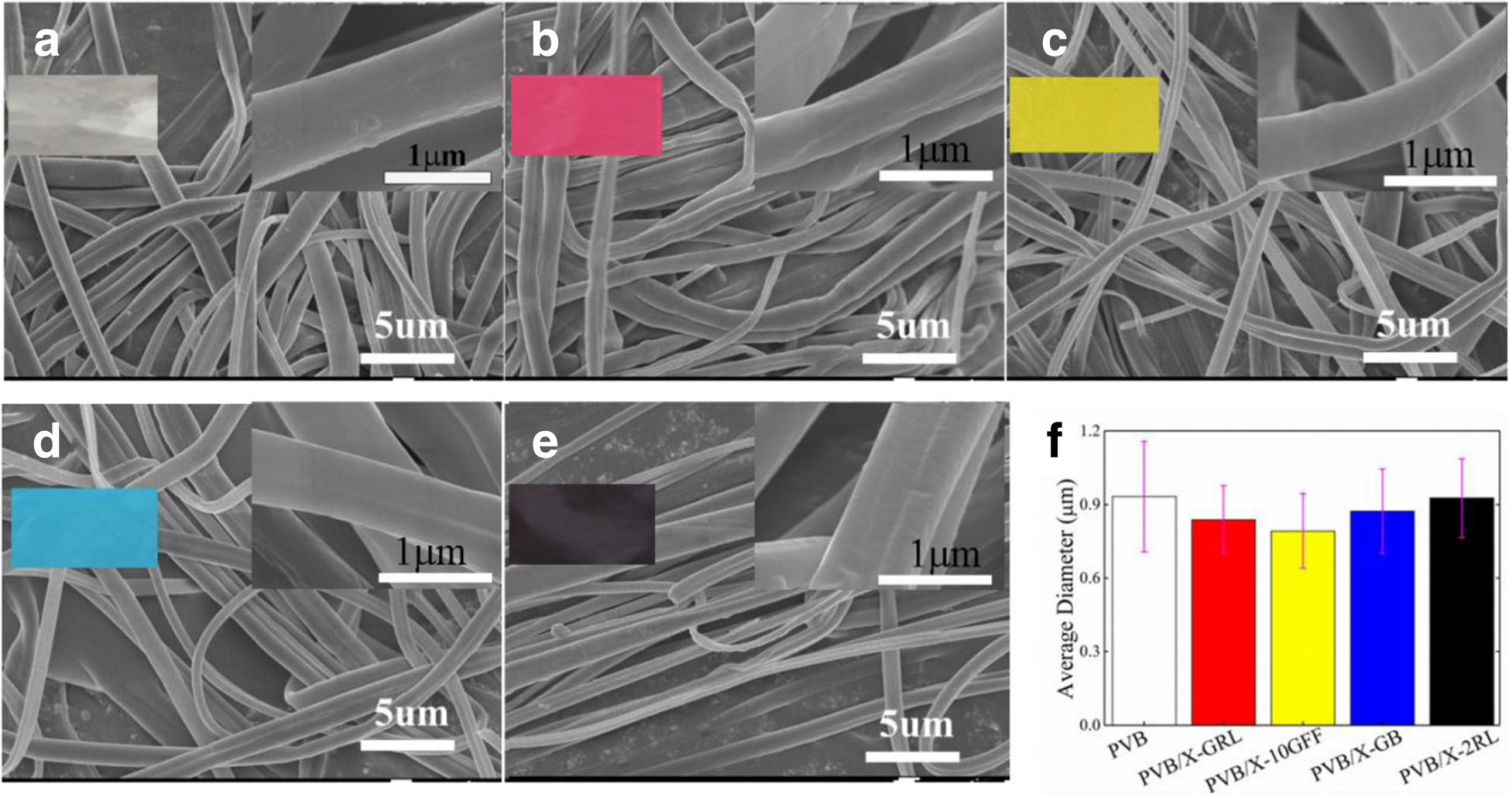
SEM images of the colorful NFMs: PVB (**a**), PVB/X-GRL (**b**), PVB/X-10GFF (**c**), PVB/X-GB (**d**), PVB/X-2RL (**e**), and the fiber diameters distributions of the prepared colorful NFMs (**f**). The *inset* SEM images in (**a**)–(**d**) are the enlarged SEM images of the corresponding colorful NFMs. The *error bars* in (**f**) suggest the degree of evenness of the different colorful NFMs diameters

The effect of cationic dyes on the microscopic morphology of obtained colorful NFMs was examined by SEM. As shown in Fig. 5a–e, the fibers of colorful NFMs are relative uniform and smooth. The inset enlarged SEM images indicate that the doping of dyes does not change the morphology of the PVB fiber a lot, which is due to the good solubility of the cationic dyes in alcohol and agrees with the former study [17]. However, the average diameter of the PVB fibers is decreased with cationic dye doping as displayed in Fig. 5f, which is due to the increasing solution conductivity as doped with cationic dyes (Additional file 1: Figure S1a). As the conductivity of the solution improved, stretching of the solution jet will increase as a result of higher level of charges carried by the solution [42]. And the increase of the solution conductivity can be attributed to the increasing positive charges ionized by cationic dyes dissolved in solution. In addition, different dyes contribute to the diameter changing different and the uniformity of the colored nanofibers can be determined by the diameter error bars as shown in Fig. 5f. Moreover, compared with the level-dyeing measurements shown in Table 1, the better uniformity of the e-spun fiber diameter and the better level-dyeing property can be obtained.

### Dye Concentration

**Fig. 6:**
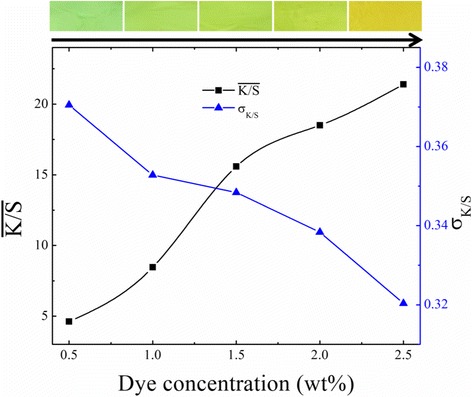
The effects of dye concentration on the color strength ($\overline{K/S}$ the left y-axis) and the level-dyeing property (σ_K/S_, the right y-axis) by taking PVB/X-10GFF as an example. The top images of color deepened NFMs corresponding to the e-spun PVB/X-10GFF NFMs with increasing dye concentration

As mentioned above, the prepared colorful cationic dye doped PVB NFMs show good level-dyeing property. For a further study, we tested the effect of dye concentration on the $\overline{K/S}$ value and σ_*K*/*S*_ by taking cationic dye X-10GFF as an example. It has been reported that $\overline{K/S}$ value shows a linear relation with increasing dye concentration [21]. As shown in Fig. 6, the color strength $\overline{K/S}$ is obviously enhanced as the increasing doping of dye, which corresponds to the color deepening of the PVB/X-10GFF NFMs in the top of Fig. 6. Moreover, the σ_*K*/*S*_ values are reduced as dye concentration increased, which suggested a better level-dyeing property with increasing doping of dye X-10GFF. The reduced σ_*K*/*S*_ values may be due to decreased fiber diameter and improved fiber uniformity with dye concentration increasing as shown in Additional file 1: Figure S2. The average diameter of PVB/X-10GFF is found to reduce from 886 to 715 nm as the doping of dye increased. The decreasing of fiber diameter can be contributed to the increasing conductivity of solutions as shown in Additional file 1: Figure S1b.

### Hydrophobicity

**Fig. 7:**
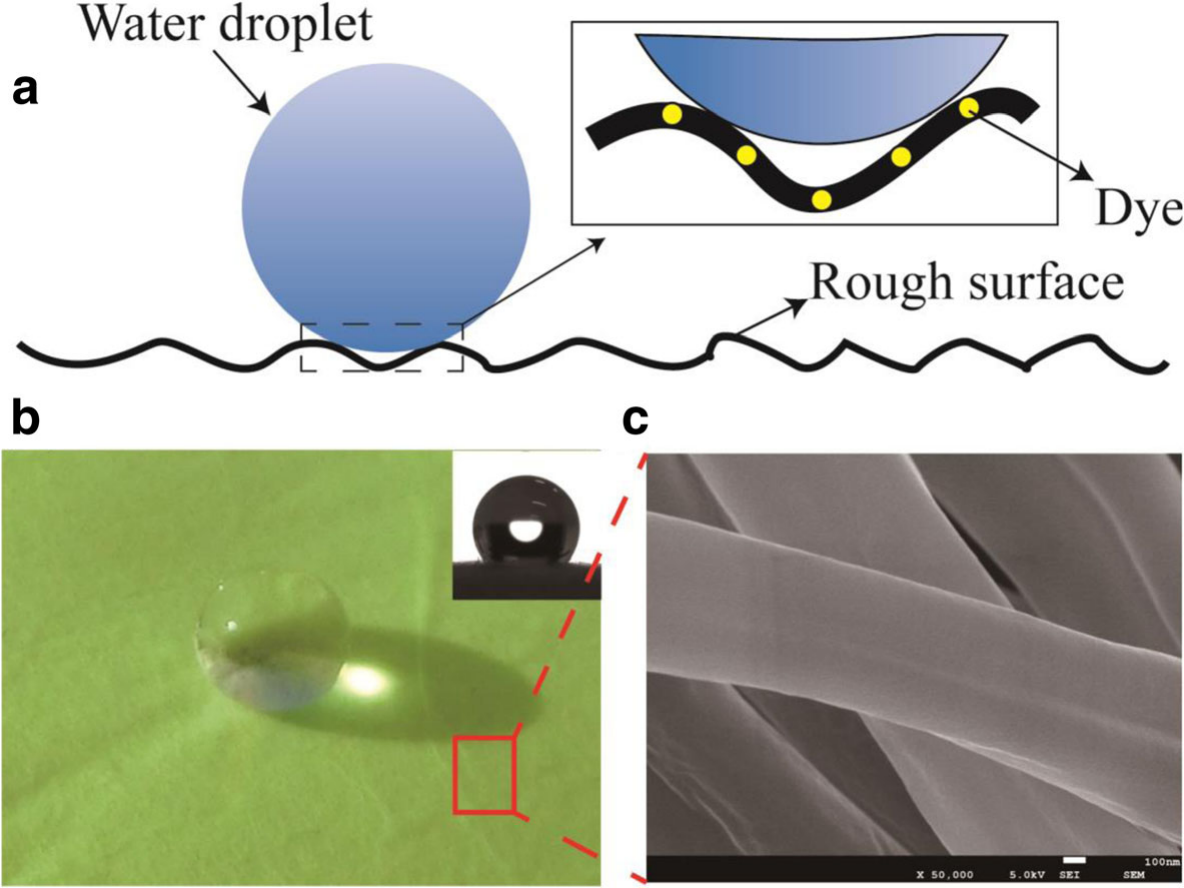
Schematic representation of the high WCA of NFM due to its rough surface (**a**), the real water droplet onto electrospun colorful PVB NFM surface (**b**), and the SEM image of colorful NFM (**c**), which suggested the dye may wrapped in the fiber as displayed in enlarge image of (**a**)

It has been reported that water contact angle (WCA) of electrospun PVB fabrous mat could reach 130°, which was much higher than WCA of the PVB film prepared by solution casting [37, 38]. The high hydrophobicity of e-spun PVB film can be attributed to the nanoscaled rough surface structure of the e-spun PVB nanofabrous mats, as shown in Fig. 7a. Accordingly, the prepared colorful PVB/dye NFMs also show high WCA, as displayed in Fig. 7b. Moreover, from Fig. 5 and Fig. 7c, we can find that the cationic dyes do not disperse on the fiber surface, that means dye molecules may wrapped in the fiber (Additional file 1: Figure S3). Consequently, water-solved dyes do not weaken the hydrophobicity of the stained NFMs.

**Fig. 8:**
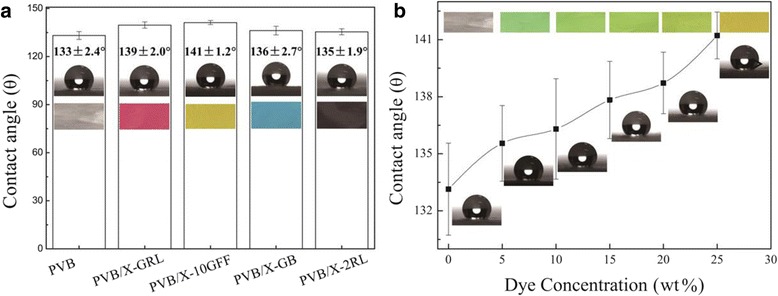
(**a**) Distribution of water contact angles on the different colorful NFMs. The *insets* are the images of colorful NFMs and the water droplet set on the surface of each colored NFM. (**b**) WCAs show different values on the surface of PVB/X-10GFF colored NFMs with different dye concentration. The upper insets are the images of e-spun PVB and stained PVB NFMs, and the lower images are the view of water droplets set on the surfaces of the corresponding NFMs. The corresponding WCAs are 133.1° ± 2.42°, 135.5° ± 1.99°, 136.3° ± 2.64°, 137.8° ± 2.03°, 138.7° ± 1.62°, and 141.2° ± 1.23°, respectively

Figure 8a shows the distribution of water contact angles on different colorful NFMs. The inset images are the stained PVB NFMs and frontier view of a water droplet set on the surfaces of PVB, PVB/X-GRL, PVB/X-10GFF, PVB/X-GB, and PVB/X-2RL; the WCAs are 133.1° ± 2.42°, 139.6° ± 1.95°, 141.2° ± 1.23°, 136.2° ± 2.72°, and 135.4° ± 1.89°, respectively. As observed, the colored PVB NFMs showed a higher surface hydrophobicity as compared with that of the pure PVB NFM. Considering the case mentioned above that the diameter of stained PVB nanofibers is smaller than that of the pure PVB ones as shown in Fig. 5f, the increasing of WCA on the colored NFMs surface is available and consistent with the former study [37]. Moreover, the smaller the fiber diameter, the higher surface hydrophobicity of the colorful NFMs exhibits.

Furthermore, the dye concentration influence on the hydrophobicity of colored PVB NFMs is also investigated. As suggested in Additional file 1: Figure S2, increasing of dye concentration may result in the decrease of the fiber diameters. Consequently, a higher WCA can be expected as dye concentration improved, just as the case shown in Fig. 8b. The WCA on the surface of e-spun pure PVB and dye-doped PVB NFMs is improved from 133.1° to 141.2° with X-10GFF dye concentration varied from 0 to 2.5 wt%. The increasing of hydrophobicity of colored e-spun PVB-based NFMs may attributed potential applications in protecting clothes and anticorrosion [43], additional with the improve color levelness as dye doping increased.

### Potential Applications for Color Printing

**Fig. 9:**
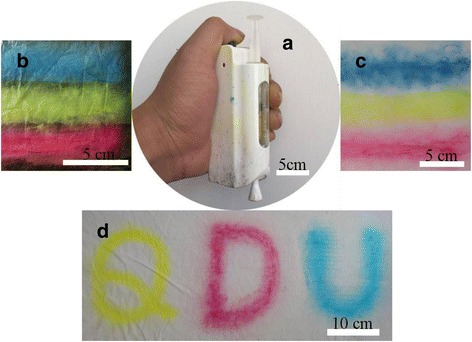
Images indicate that CSE process and as-spun colorful PVB NFM can show potential application for color printing, where **a** shows the CSE can be processed by a battery portable electrospinning apparatus, the as-spun colorful PVB NFMs can be printed onto aluminum foil substrates (**b**), wall (**c**), and fabrics (**d**)

As mentioned above, uniform and hydrophobic colorful PVB NFMs can be obtained by the CSE process. Moreover, by using a battery-operated portable electrospinning apparatus (BOEA) [44], the CSE process can be worked for color printing freely onto different objects, as displayed in Fig. 9. Taking the BOEA as a tool for CSE process (Fig. 9a), colorful PVB NFMs can be directly e-spun onto Al foil, wall, and fabrics, as shown in Fig. 9b–d, respectively (the adhension between membranes and these substrates was shown in Additional file 1: Figure S4). Thanks to the hydrophobicity of these as-spun colorful PVB NFMs, potential applications for waterproof textiles, wallpapers, and anticorrosive coating/painting can be expected. Especially, color printing text or pictures with the BOEA and CSE process can be realized as suggested in Fig. 9d, which may attract interests for teaching demonstration.

## Conclusions

In summary, various cationic dye colored PVB nanofibrous membranes have been fabricated successfully by a colored solution electrospinning process. The color measurements indicate that the as-spun colorful membranes have excellent level-dyeing property and color stability, which promise the colored solution electrospinning is a useful and efficient way to prepare colorful nanofibrous membranes for industrial application. It is found that the dye doping and the increase of dye concentration could decrease fiber diameters of the stained PVB/cationic dye membranes. Consequently, the obtained colorful PVB-based fibrous membranes exhibit better level-dyeing property and higher hydrophobicity. The colored solution electrospinning and as-spun uniform hydrophobic colored PVB nanofibrous membranes may have potential applications in color printing and anticorrosive coating/painting.

## Additional file

Additional file 1: Supplementary Materials. (DOCX 3474 kb)
