# Supplementary material for: Colorful Hydrophobic Poly(Vinyl Butyral)/Cationic Dye Fibrous Membranes via a Colored Solution Electrospinning Process
Source: Nanoscale Res Lett. 2016 Dec 5;11:540. doi: 10.1186/s11671-016-1763-4 (PMC5138176; doi:10.1186/s11671-016-1763-4)
Supplement: Supplementary file 1 — Supplementary Materials. (DOCX 3474 kb) [file 11671_2016_1763_MOESM1_ESM.docx]

**Supplementary Materials**

**Colorful hydrophobic poly(vinyl butyral)/cationic dye fibrous membranes via a colored solution electrospinning process**


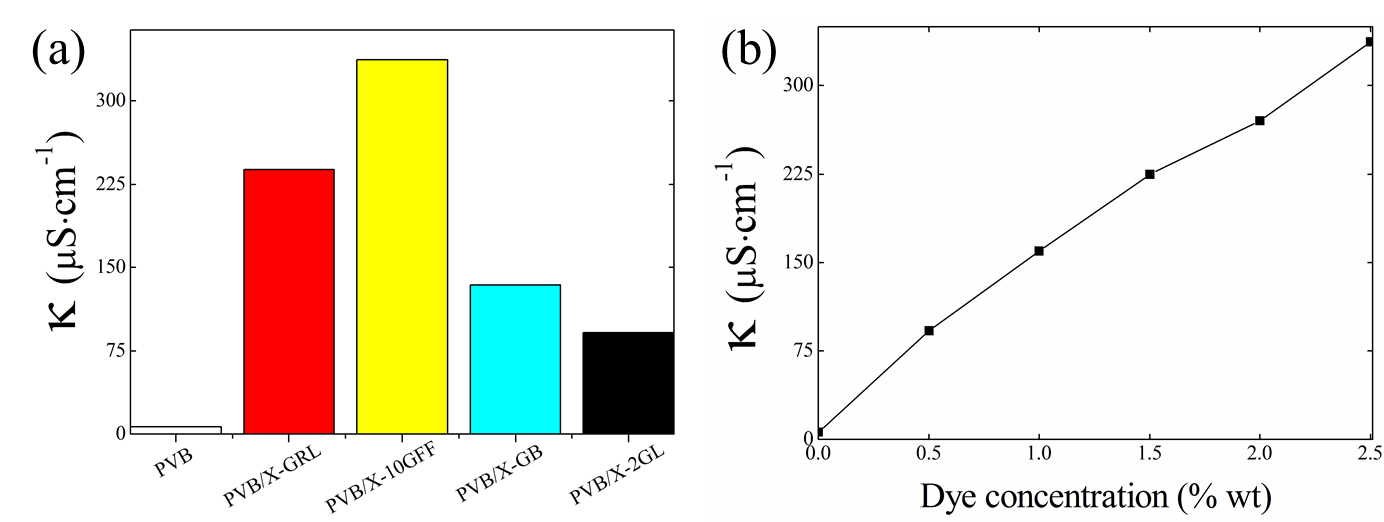


**Figure S1** Conductivities of polymer solutions (a) different dye dissolved in PVB solutions with dye concentration of 2.5 wt%; (b) conductivities of PVB/X-10GFF solutions with different X-10GFF concentrations.

**
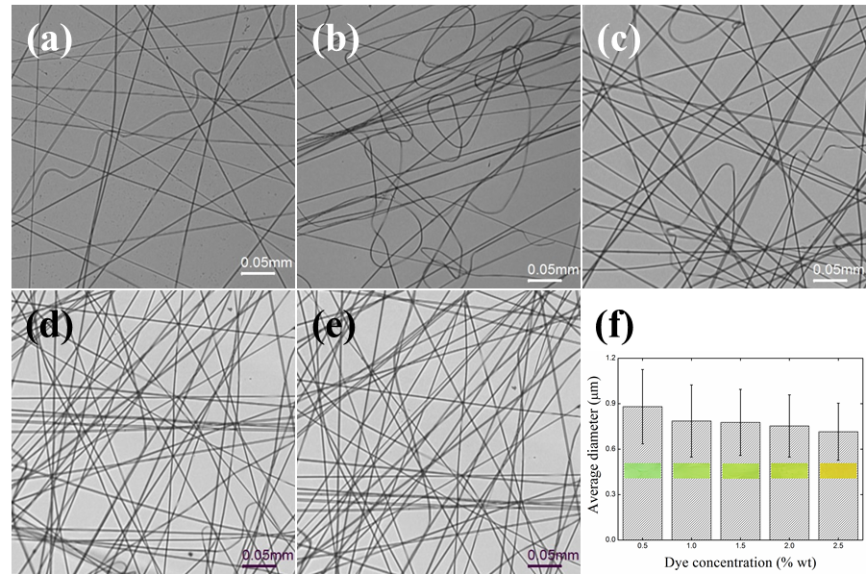
**

**Figure S2** Optical images of the PVB/X-10GFF NFMs with different dye concentration: 0.5 wt% (a), 1.0 wt% (b), 1.5 wt% (c), 2.0 wt% (d), 2.5 wt% (e), and the fiber average diameter distributions (f). The inset colorful images in (f) are the corresponding PVB/ X-10GFF colorful NFMs with different dye concentrations, and the error bars can measure the degree of uniform of these PVB/ X-10GFF fibers.


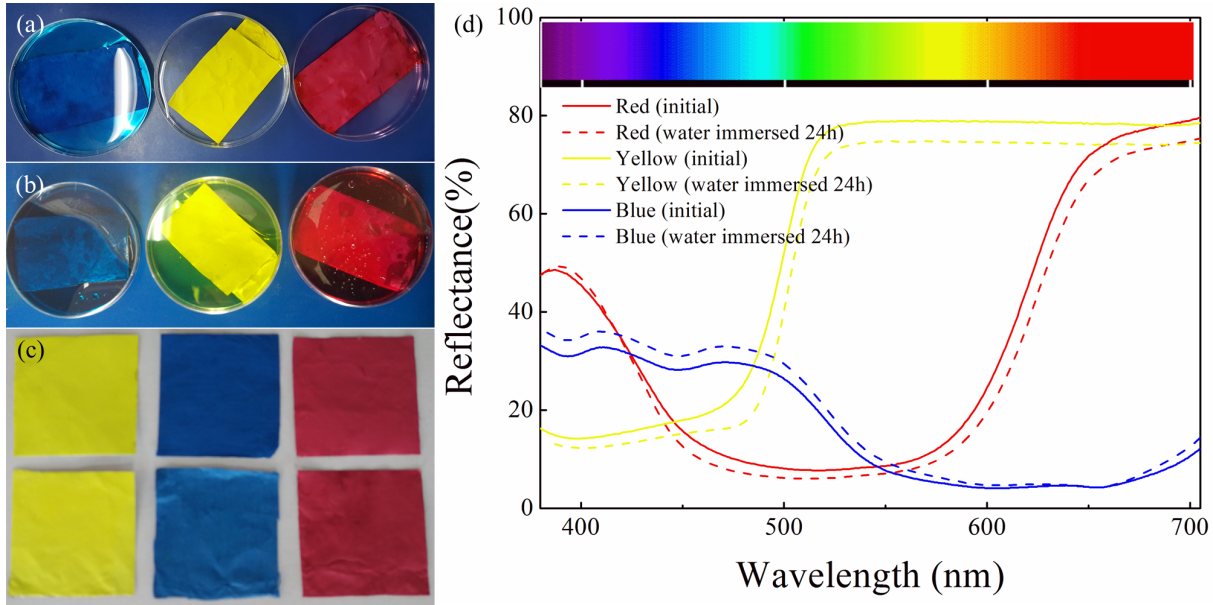


**Figure S3** The as-spun colorful membranes immersed in water (a), after 24h (b), the images of colorful membranes before and after immersed in water (c), and the UV-Vis diffuse reflectance spectroscopy of these membranes (d).


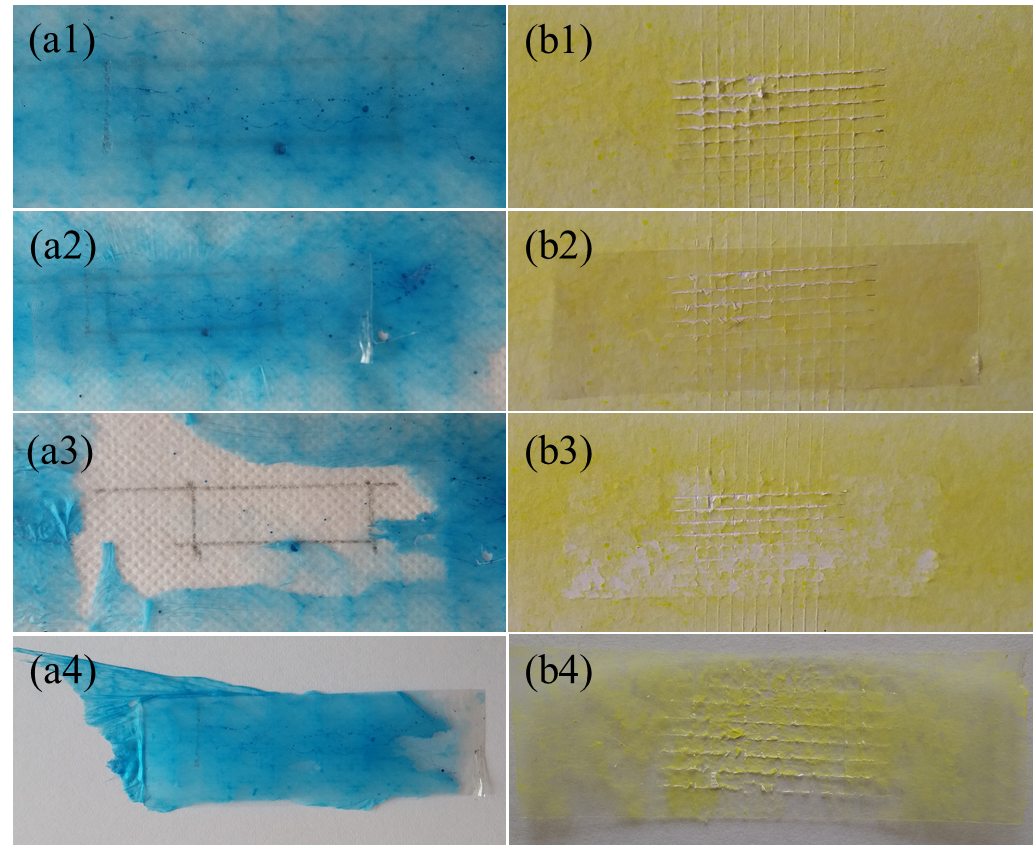


**Figure S4** cross-cut adhesion method is used to measure the adhension between the as-spun colorful membrane and the substrates. Firstly, grids were cut on the membranes (a1, b1), then 3M indicating adhesive tape were tagged to the grid region (a2, b2), removing the tape, some membranes can be tear off from the substrates (a3, a4, b3,b4). By counting the grids with membranes left (a3,b3), the adhension can be measured qualitatively.
